# A Hybrid Method Applied to Improve the Efficiency of Full-Waveform Inversion for Pavement Characterization

**DOI:** 10.3390/s18092916

**Published:** 2018-09-03

**Authors:** Jingwei Zhang, Shengbo Ye, Li Yi, Yuquan Lin, Hai Liu, Guangyou Fang

**Affiliations:** 1 School of Electronic, Electrical and Communication Engineering, University of Chinese Academy of Sciences, Beijing 100149, China; linyuquan16@mails.ucas.edu.cn; 2 Institute of Electronics, Chinese Academy of Sciences, Beijing 100190, China; sbye@mail.ie.ac.cn (S.Y.); gyfang@mail.ie.ac.cn (G.F.); 3 Key Laboratory of Electromagnetic Radiation and Sensing Technology, Chinese Academy of Sciences, Beijing 100190, China; 4 Fukushima Renewable Energy Institute, AIST (FREA), Fukushima 963-0298, Japan; li.yi@aist.go.jp; 5 School of Civil Engineering, Guangzhou University, Guangzhou 510006, China

**Keywords:** ground penetrating radar (GPR), hybrid method, multilayer perceptrons (MLPs), thin layer

## Abstract

Ground penetrating radar (GPR), as a nondestructive testing tool, is suitable for estimating the thickness and permittivity of layers within the pavement. However, it would become problematic when the layer is thin with respect to the probing pulse width, in which case overlapping between the reflected pulses occurs. In order to deal with this problem, a hybrid method based on multilayer perceptrons (MLPs) and a local optimization algorithm is proposed. This method can be divided into two stages. In the first stage, the MLPs roughly estimate the thickness and the permittivity of the GPR signal. In the second stage, these roughly estimated values are used as the initial solution of the full-waveform inversion algorithm. The hybrid method and the conventional global optimization algorithm are respectively used to perform the full-waveform inversion of the simulated GPR data. Under the same inversion precision, the objective function needs to be calculated for 450 times and 30 times for the conventional method and the hybrid method, respectively. The hybrid method is also applied to a measured data, and the thickness estimation error is 1.2 mm. The results show the high efficiency and accuracy of such hybrid method to resolve the problem of estimating the thickness and permittivity of a “thin layer”.

## 1. Introduction

Sounding pavement layers is classically performed using standard ground penetrating radar (GPR) [1,2], whose vertical resolution is bandwidth dependent. For this purpose, the pavement is assumed to be horizontally stratified. The vertical structure of pavement can then be deduced from GPR profiles by means of echo detection and amplitude estimation. Echo detection provides time delay (TDE) associated with each interface, whereas amplitude estimation is used to retrieve the permittivity of each layer. When a layer is thick with respect to the probing pulse width, the echoes respectively from the surface and from the bottom of it can be easily detected. However, when the case comes to a thin layer, the aforementioned two echoes become overlapped. In this case, estimating TDE and amplitude of each echo becomes difficult. Herein, it is referred as the “thin layer problem” [3]. This article focus on dealing with the “thin layer problem”.

In order to address the “thin layer problem”, Li [4] proposed a method based on independent component analysis (ICA), which performed a blind separation of the overlapped echoes. In the basic assumption of the ICA model, the components of the mixed sources are mutually independent. However, in the GPR signal the echoes associated with interfaces are mutually correlated [1]. Le Bastard et al. [5] studied the performance of several high-resolution and super-resolution methods for separating the overlapped echoes in the GPR signal. The results show that these methods are model dependent, and their performance is related to the simplified electromagnetic hypothesis sustained by the GPR data model [6]. For example, the multiple signal classification (MUSIC) algorithm has a super-resolution for TDE estimation, but its performance can be significantly degraded if multipath effects are present in the signal. In the pavement inspection, the received echoes are simple time-shifted and attenuated replicas of the emitted radar pulse, which is exactly one manifestation of multipath effects. According to the recent literatures [7,8], the full-waveform inversion method seems to be a promising approach to solve the “thin layer problem”. However, the performance of the full-waveform inversion method depends on the accuracy of the forward model and the effect of the optimization algorithm. Lambot et al. [7] proposed a method to model a GPR system in far field conditions. In their method, the GPR including the effects of antenna is modeled by a linear equation system. This physically based approach has been demonstrated to be significantly accurate on emulating GPR signal and retrieving medium properties [7]. Nevertheless, the inverse problem is always ill-posed, and many local minima are present in the solution space. Therefore, the inversion process needs to be resorted to global optimization algorithms (i.e., multi-scale coordinate search (MCS) algorithm [9], differential evolution (DE) algorithm [10]), which are computationally intensive and inefficient. In order to improve the efficiency of the inversion process, De Coster et al. [8] elaborated a lookup table (LUT), i.e., a matrix in which signals pre-computed for different parameter combinations are stored. Although this technique can greatly reduce the computational cost in the inversion process, it does not improve the efficiency of the global optimization algorithm. Furthermore, this technique increases the cost of GPR system. Machine learning (ML) algorithms have been introduced into the field of GPR in recent years [11,12,13], and some of them have shown promising results. Rodriguez et al. [14] proposed a method based on principal component analysis (PCA) and Neural Networks (NNs) to interpret the data compiled in GPR-based geological surveys. The results show that this approach is promising in applications where complete reconstruction of the subsoil is required. Xie et al. [15] used support vector machine (SVM) to recognize the voids in GPR images of reinforced concrete structure and reached a maximum accuracy of 96%. Shangguan et al. [16] used NNs to estimate the density of asphalt pavement. The results show that the density information can be accurately obtained by this approach. Kilic [17] used NNs to detect the beam position in GPR data and the results show the proposed approach is promising in this application. In addition to the aforementioned applications, ML algorithms are also potential approaches to solve the “thin layer problem”. De Bastard et al. [18] successfully trained an estimator with the support vector regression (SVR) algorithm to estimate the TDE of the echoes and dielectric constants of the media. However, in the model of their training data, the Dirac function is used as the emitted pulse, which is too ideal to be implemented in practical applications.

In this article, a hybrid method consisting of multilayer perceptrons (MLPs) and a local optimization algorithm is proposed to estimate the thickness and permittivity of the “thin layer”. Since the layer thickness and permittivity of the pavement are all within limited ranges, therefore, these parameter ranges can be divided into several intervals. A MLPs network is trained to classify the received GPR signal into specific combination of the parameter intervals according to pre-defined rules. Then, midpoint values of the intervals under the combination are used as the initial solution of the full-waveform inversion algorithm. In order to make the proposed method suitable for real-world situations, the training data for the MLPs network are simulated with the GPR model proposed by Lambot. This consideration is of practical significance, for example, the thickness of the surface layer of hot-mix asphalt (HMA) pavement is always within 4 cm [19], and the permittivity of HMA is also within a limited range from 4 to 8 according to previous literatures [6,19]. This proposed hybrid method is applied to the simulated data and measured data to validate the performance.

Section 2 briefly introduces the principles of the GPR modeling method, the MLPs, the full-waveform inversion, and the proposed hybrid method. Section 3 describes the implementation of the proposed hybrid method, and the proposed method is applied to the simulated data and measured data to verify its performance.

## 2. Principles of the Basic Methods

### 2.1. Principle of the GPR Modeling Method

In the hybrid method, the MLPs are trained with the GPR data simulated by the GRP model proposed by Lambot et al. In their method, the GPR in the far-field region can be modeled by a linear equation system under the assumption that the electric field measured by the antenna tends to be a plane wave. The GPR signal can accordingly be calculated by [7]:

(1) $$S_{11\;}(\omega )=\frac{b(\omega )}{a(\omega )}=H_{i}(\omega )+\frac{H(\omega )G_{xx}^{0}(\omega )}{1-H_{f}(\omega )G_{xx}^{0}(\omega )}$$

where, *b* and *a* are the backscattered waves and the incident waves, respectively, $H_{i}$ is the mutual coupling coefficient between the transmitting and receiving antenna, H is the transmitting-receiving transfer function accounting for antenna gain and phase delay, $H_{f}$ is the feedback loss accounting for the multiple reflections occurring between the antenna and pavement, and $G_{xx}^{0}$ is the zero-offset Green’s function of the pavement layers. The coefficients of the GPR model can be determined by solving the system of Equation (1) for different model configurations (denoted *n*, ranging from 1 to *K*). Same as that in [7], the well-defined model configurations with the antenna situated at different heights above a large copper plate is used. Therefore, the Green’s functions $G_{xx,n}^{0}(\omega )$ can be calculated, while the functions $S_{11,n}(\omega )$ can be readily measured. Then, the system of Equation (1) can be rewritten as

(2) $$S_{11,n\;}=H_{i}+S_{11,n}\cdot G_{xx,n}^{0}\cdot H_{f}+G_{xx,n}^{0}\cdot (H-H_{i}\cdot H_{f}).$$

The linear system of (2) can be written in matrix form as [7]

(3) b=Ax 

where

(4) $$b={\left[S_{11,1\;},\ldots ,S_{11,n},\ldots ,S_{11,K}\right]}^{T}$$

(5) $$A=\left[\begin{array}{ccc} 1 & S_{11,1\;}G_{11,1}^{0} & G_{11,1}^{0}\\ \vdots & \vdots & \vdots \\ 1 & S_{11,K}G_{11,K}^{0} & G_{11,K}^{0} \end{array}\right]$$

(6) $$x={\left[H_{i},H_{f},H-H_{i}H_{f}\;\right]}^{T}.$$

Using the least squares approach, the vector x can be easily calculated as

(7) $$x={\left(A^{H}A\;\right)}^{-1}A^{H}b$$

where the superscript *H* denotes the Hermitian operation. More detailed information of the GPR modeling method is in [7].

### 2.2. Principle of Multilayer Perceptrons

**Figure 1:**
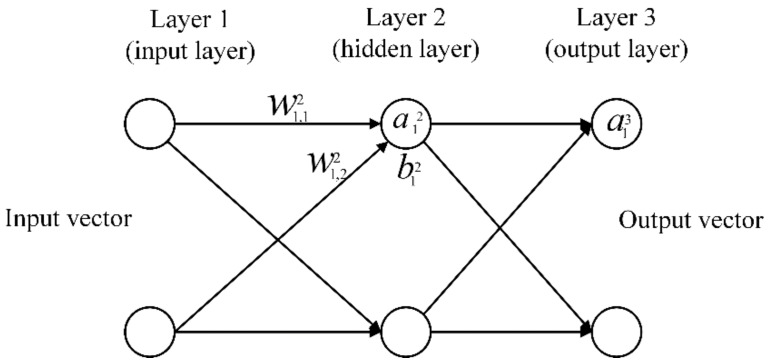
The basic structure of the MLPs. There is only one hidden layer in the MLPs.

When the GPR model is calibrated, it can be used to simulate the received GPR data under various pavement models to train the multilayer perceptrons (MLPs). MLPs network is one type of multilayer feedforward neural networks. It consists of an input layer, one or more hidden layers, and an output layer. MLPs have the ability to approximate any continuous function. The number of neurons in each hidden layer of MLPs is heuristically set according to the problem to be processed. The more neurons there are, the stronger approximation ability of the network, and the greater risk of overfitting [20]. However, the number of neurons in the output layer is determined by the dimensions of the problem being processed. For example, for a multi-class classification problem, the number of neurons in the output layer is equal to the number of categories to be classified. Figure 1 shows the MLPs with one hidden layer.

In Figure 1, $a_{j}^{l}$ denotes the activation of the jth neuron of the lth layer, $b_{j}^{l}$ denotes the bias of the jth neuron of the lth layer, $w_{jk}^{l}$ denotes the weight connecting the jth neuron of the lth layer and the kth neuron of the (l−1)th layer. With these notations, the activation value of the jth neuron of the lth layer can be associated with the activation values of the (l−1)th layer by the following equation

(8) $$a_{j}^{l}=\sigma (\sum_{k}w_{jk\;}^{l}a_{k}^{l-1}+b_{j}^{l})$$

where the summation is performed on all *k* neurons in the (l−1)th layer, and σ denotes the activation function. In the classification scenario, the MLPs has to perform a mapping from the input space into a finite set of class Y={1,…,Lab}, where Lab is the number of classes. In the training phase, the parameters of the MLPs are determined from a finite training set

(9) $$TRAIN=\left\{\left(V^{\gamma },y^{\gamma },\right)|V^{\gamma }\in {\mathbb{R}}^{d},y^{\gamma }\in Y,\gamma =1,\ldots ,\mathrm{TR}\right\},$$

here each training data $V^{\gamma }$ is labelled with its class membership $y^{\gamma }$, and TR is the number of training data. To determine the parameters of the network, MLPs are trained in a supervised manner with a highly popular algorithm known as the error back-propagation algorithm [21]. This algorithm is based on the error-correction learning rule. Most often the mean square error (MSE) is minimized [22]

(10) $$\mathrm{MSE}=\frac{1}{Lab\cdot TR\;}\sum_{\gamma =1}^{TR}\sum_{j=1}^{Lab}{\left(y_{j}^{\gamma }-a_{j}^{out,\gamma }\right)}^{2}$$

(11) $$y_{j}^{\gamma }=\{\begin{array}{cc} 1 & y^{\gamma }=j\;\\ 0 & y^{\gamma }\neq j \end{array}$$

where $y_{j}^{\gamma }$ means the desired value of the jth network output for the γth instance, $a_{j}^{out,\gamma }$ denotes the actual value of the jth network output for the γth instance. In the training process of MLPs, the weights and biases are updated in the manner of gradient descent

(12) $${\hat{w}}_{jk\;}^{l}=w_{jk}^{l}-\eta \cdot \frac{\partial MSE}{\partial w_{jk}^{l}}$$

(13) $${\hat{b}}_{j}^{l}=b_{j}^{l}-\eta \cdot \frac{\partial MSE\;}{\partial b_{j}^{l}}$$

where ${\hat{w}}_{jk}^{l}$ and $w_{jk}^{l}$ are respectively the updated weight value and the weight value before the update in each iteration, ${\hat{b}}_{j}^{l}$ and $b_{j}^{l}$ are respectively the updated bias and the bias before the update in each iteration, η is the learning rate, and $\frac{\partial \cdot }{\partial \cdot }$ denotes the partial derivative operation. The training process of MLPs is sensitive to the learning rate. If the learning rate is too large, the error function will oscillate during the descent process. If the learning rate is too small, the error function will drop slowly which reducing the efficiency of training. A highly popular way is to make the learning rate exponentially decrease from a large value with the number of iterations of training. In order to improve the training efficiency of MLPs, some other techniques are often introduced in training process, such as dropout, regularization, bath normalization et al., and more detail information about MLPs can be found in [23,24]. In the inference phase, an instance without label is fed to the network, and the categorization is performed by assigning the input vector V the class of the output neuron with maximum activation:

(14) $$\mathrm{class}\left(V\right)=\underset{j\in \left\{1,\ldots ,Lab\right\}}{\arg\max}\left[a_{j}^{out}(V)\right].$$

### 2.3. Principle of the Hybrid Method

**Figure 2:**
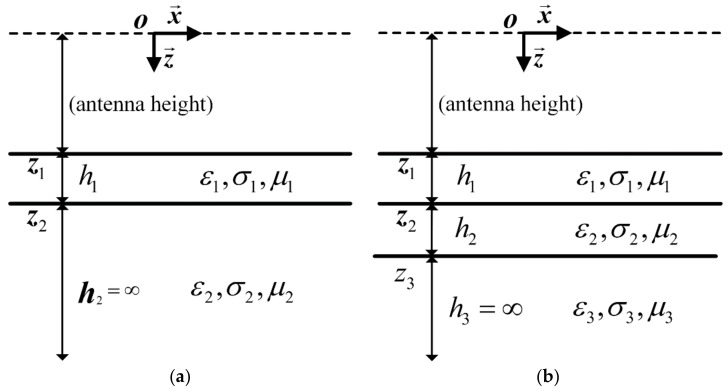
Pavement models used to simulate the training data for MLPs. (**a**) The two layer pavement model. (**b**) The three layer pavement model. The first layer of both the two models is a thin layer with respect to the pulse width. The second layer of the three layer pavement model is a thick layer with respect to the pulse width. The electric conductivities of all layers are assumed to be negligible.

The proposed hybrid approach is a method based on supervised ML algorithm. In order for the ML algorithm to have a good effectiveness in practical applications, the training data need to cover all possible situations as much as possible. In pavement inspection, the pavement is often assumed to be horizontally stratified under the antenna footprint. Under this condition, the GPR signals emulated by the Lambot’s far-field model have a high degree agreement with the measured data [7]. Therefore, in the proposed hybrid method, the far field model is used to simulate the training data. In the simulation process, each simulated GPR signal is determined by the height of the antenna and the characteristic parameters of the pavement model. In pavement inspections, the antenna is always located at a height with a small variation due to the unevenness of the pavement surface. Hence, when simulating the training data, the height parameter of the antenna is set to a random value within a small variation range. In addition, two kinds of pavement models are used in the simulation (Figure 2). The first kind pavement model consists of two layers, the first layer of it being a thin layer, and the second layer being an infinite one. The second pavement model consists of three layers, the first layer of it being a thin layer, the second being a thick layer with respect to the probing pulse width, and the third one being an infinite layer. In order to estimate the thickness of the first layer and the permittivities of the first two layers, only the echoes from the first two interfaces need to be matched in the full-waveform inversion. Therefore, this three-layer pavement model can cover all situations where the number of layers is greater than two. Here, the two kinds of pavement models are used to generate training data set, which can ensure that the ML algorithm learns the essential features in these data and enhances the robustness of the prediction model.

**Figure 3:**
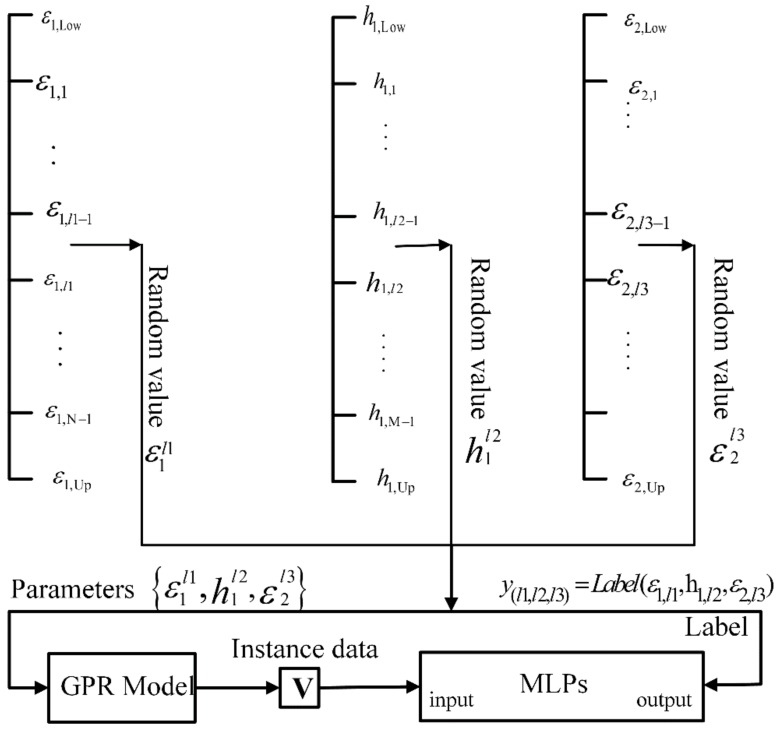
Process of training the MLPs using simulated GPR data. The ranges of the thickness of the first layer and the ranges of the permittivities of the first two layers are divided into small intervals. Each intervals combination $\left(\varepsilon_{1,l1},h_{1,l2},\varepsilon_{2,l3}\right)$ can be assigned a label $y_{\left(l1,l2,l3\right)}$. Under this intervals combination, parameters combination $\left\{\varepsilon_{1}^{l1},h_{1}^{l2},\varepsilon_{2}^{l3}\right\}$ can be randomly generated. The parameters combination is used to simulate GPR data, and the GPR data is rearranged into the instance V. Then the instance V and the corresponding label $y_{\left(l1,l2,l3\right)}$ are used to train the MLPs.

According to the above descriptions, it is assumed that the thickness of the first layer of the pavement is within the range of $\left[h_{1,\mathrm{Low}},h_{1,\mathrm{Up}}\right]$, and the permittivity of the pavement is in the range of $\left[\varepsilon_{\mathrm{Low}},\varepsilon_{\mathrm{Up}}\right]$. In order to improve the efficiency of parameter search in the process of the full-waveform inversion, these parameter ranges are divided into small intervals. For example, the permittivity range is divided into $\left[\varepsilon_{Low},\varepsilon_{1}\right)$, $\left[\varepsilon_{1},\varepsilon_{2}\right)$, …, $\left[\varepsilon_{i-1},\varepsilon_{i}\right)$, …, $\left[\varepsilon_{N-1},\varepsilon_{\mathrm{Up}}\right]$ as N intervals, where $\Delta \varepsilon =\varepsilon_{i}-\varepsilon_{i-1}$. Same as the previous operation, the thickness range of the first layer is divided into $\left[h_{1,Low},h_{1,1}\right)$, …, $\left[h_{1,i-1},h_{1,i}\right)$, …, $\left[h_{1,M-1},h_{1,\mathrm{Up}}\right]$ as M intervals, where $\Delta h_{1}=h_{1,i}-h_{1,i-1}$. With these small parameter intervals, all possible properties combinations of the first two layers of the pavement can be divided into a limited number of categories, which corresponds to a multi-class classification problem. For each properties interval combination, a label y can be assigned according to $y_{\left(l1,l2,l3\right)}=Label(\varepsilon_{1,l1},h_{1,l2},\varepsilon_{2,l3})$, where, *l*1, *l*2, *l*3 are respectively the index number of intervals in original ranges, Label(⋅) denotes a mapping operation. For the class $y_{\left(l1,l2,l3\right)}$, a number of random values are generated according to the uniform distribution over the parameter intervals belonging to it. Then, these randomly generated parameter combinations are used to simulate GPR signals, and these simulated GPR data belong to the class $y_{\left(l1,l2,l3\right)}$. It should be noted that for the three-layer pavement model, the thickness of the second layer can be set to a random value within a meaningful range, and the permittivity of the third layer can be simply set to a fixed value. However, the thickness of the second layer and the permittivity of the third layer do not affect the classification results. Since the GPR signal obtained by the simulation is in frequency domain form, it is rearranged as follow:

(15) $$V={\left[ℜ\left[S\left(f_{1}\;\right)\right],ℑ\left[S\left(f_{1}\right)\right],\ldots ,ℜ\left[S\left(f_{k}\right)\right],ℑ\left[S\left(f_{k}\right)\right],\ldots ,ℜ\left[S\left(f_{Q}\right)\right],ℑ\left[S\left(f_{Q}\right)\right]\right]}^{T}$$

where $ℜ\left[S\left(f_{k}\right)\right]$ and $ℑ\left[S\left(f_{k}\right)\right]$ are respectively the real part and imaginary part of the radar data at frequency $f_{k}$, *Q* is the number of frequency points of the GPR data, and vector V is the instance used to train the MLPs. Figure 3 shows the MLPs training process starting from the simulated data. During the phase of inference, an input GPR signal is predicted to a class by MLPs. Then, the midpoint values of all parameter intervals under this class are used as the initial solution for the full-waveform inversion of this GPR signal. If the classification result is accurate, the maximum distance between each component of the initial solution and its corresponding global optimal solution is δ/2, where δ is the length of the interval. Therefore, if δ is small, the initial solution is close to the global optimal solution, so an efficient local optimization algorithm can meet the requirement of the full-waveform inversion, i.e., the Levenberg-Marquardt algorithm.

### 2.4. Principle of the Full-Waveform Inversion

**Figure 4:**
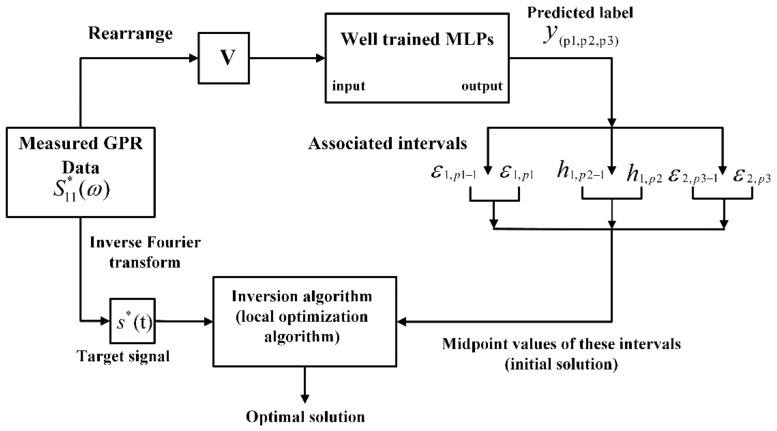
Process of the inversion starting from the initial solution provided by the MLPs. The measured GPR data $S_{11}^{*}(\omega )$ on one hand is transformed into the target signal $s^{*}(t)$, and on the other hand is rearranged into the form of V. Then, V is fed to the well trained MLPs, and a label $y_{\left(p1,p2,p3\right)}$ is predicted. According to the preset rule, the label $y_{\left(p1,p2,p3\right)}$ is associated with the intervals combination $\left(\varepsilon_{1,p1},h_{1,p2},\varepsilon_{2,p3}\right)$, and the midpoint values of these intervals are used as the initial solution. Then, the initial solution and the target signal $s^{*}(t)$ are used to perform the inversion.

When the MLPs network is well trained, it can be used to classify the measured GPR data into a specific class $y_{\left(p1,p2,p3\right)}$. Since the class $y_{\left(p1,p2,p3\right)}$ is associated with the parameter intervals combination $\left\{\left[\varepsilon_{1,p1-1},\varepsilon_{1,p1}\right),\left[h_{1,p2-1},h_{1,p2}\right),\left[\varepsilon_{2,p3-1},\varepsilon_{2,p3}\right)\right\}$, the midpoint values of the these intervals under this combination are used as the initial solution for the full-waveform inversion. The full-waveform inversion is a nonlinear optimization problem which consists in finding the parameter vector $z=\left[\varepsilon_{u},\sigma_{u},h_{u}\right]$, *u* = 1, …, R, so that an objective function obj(z) is minimized [7]. Since the GPR only receives signals in a limited time range, the full-waveform inversion is generally performed in the time domain. Therefore, the objective function is accordingly defined as follows:

(16) $$\mathrm{obj}(z)=\frac{{\left|s^{*}(t)-s(t)\;\right|}^{T}\left|s^{*}(t)-s(t)\right|}{\left\|s^{*}(t)-s(t)\right\|}$$

(17) $$s^{*}(t)=\mathrm{IFFT}\left[S_{11\;}^{*}(\omega )\right]$$

(18) $$s(t)=\mathrm{IFFT}\left[S_{11\;}(\omega ,z)\right]$$

where $s^{*}(t)$ is the vector containing the time domain target signal for inversion, s(t) is the vector containing the time domain signal simulated by the unknown parameter vector z during the inversion process, IFFT[⋅] denotes the inverse Fourier transform, $S_{11}^{*}(\omega )$ denotes the measured GPR data, $S_{11}(\omega ,z)$ denotes the simulated GPR data, ‖⋅‖ denotes the norm of the vector, and the superscript *T* denotes the transpose operation. Since the inversion is only performed on the thickness of the first layer and the dielectric constants of the first two layers, it is necessary to properly select the time range t of the target signal so that only the echoes from the first two interfaces are included in the target signal. Within the selected time range, the target signal can be approximated as the GPR response of the two-layer pavement. Therefore, in the inversion process, the simulated GPR data $S_{11}(\omega ,z)$ is obtained by using the two-layer pavement model. In most electromagnetic inverse problems, this objective function is highly nonlinear and is characterized by an oscillatory behavior and a multitude of local minima [7]. This complex topography necessitates the use of a robust global optimization algorithm. However, the global optimization algorithms are often inefficient. In our method, the initial solution is provided by the MLPs instead of randomly selected. Since the initial solution provided by the MLPs is close to the global optimal solution, the inversion can be performed by using an efficient local optimization algorithm. Figure 4 shows the process of the inversion starting from the initial solution provided by the MLPs.

## 3. Results and Discussion

### 3.1. Numerical Experiment of the Hybrid Method

**Figure 5:**
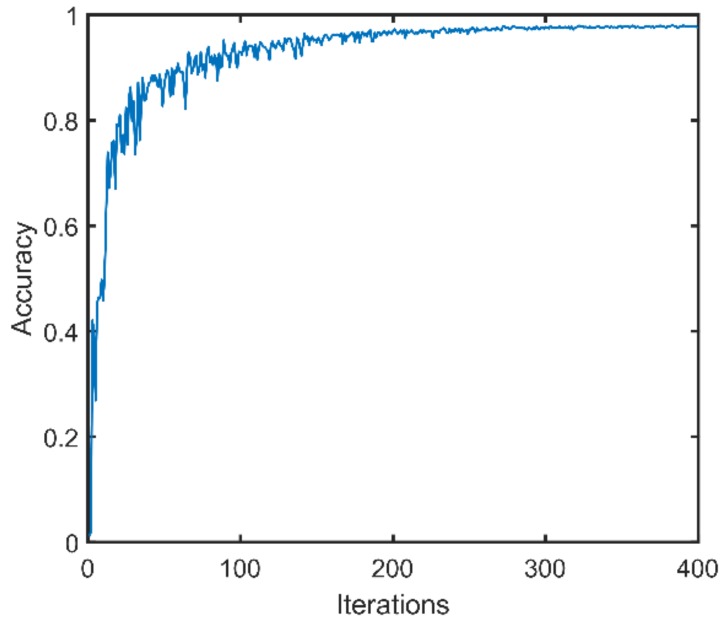
The classification accuracy of the MLPs on the test set after each iteration. The maximum classification accuracy is 98.6%, which means a GPR data can be accurately classified to the specific class according to the preset rules.

This section aims at illustrating that the proposed hybrid method can improve the efficiency of the full-waveform inversion. First of all, a mono-static GPR composed of a homemade horn antenna and a portable vector network analyzer (VNA) is modeled with the method introduced in Section 2.1. The portable VNA model is produced by COPPER MOUNTAIN TECHNOLOGIES (S5048) [25], and its operating frequency ranges from 20 kHz to 4.8 GHz. The dimensions of the homemade horn antenna are 22 cm length and 16 cm × 30 cm aperture area. Its nominal frequency is 0.75–4.5 GHz, and its isotropic gain ranges from 6–14 dBi. In the experiment, the mono-static GPR operates on the frequency range from 0.9 GHz to 3.5 GHz with 108 frequency points. To calibrate the mono-static GPR model, measurements were performed with antenna at 41 different heights from 0.4 m to 0.6 m over a 2.5 m × 2.5 m copper sheet. During the preparation of the training data, white noise is added to the simulated GPR data with a moderate signal to noise ratio (SNR) i.e., 20 dB. The antenna height is in the range from 0.45 m to 0.47 m. The thickness of the first layer is within the range from 2 cm to 5 cm, the relative permittivity of the first layer is within the range from 4 to 7, and the relative permittivity of the second layer is within the range from 5 to 8. The dielectric constant of the second layer is set larger than that of the first layer because the second layer has a larger degree of compaction, which is proportional to the dielectric constant [26]. According to the descriptions in Section 2.4, the thickness range of the first layer ($h_{1}\in [2,5]$) is divided into [2,2.5), [2.5,3), [3,3.5), [3.5,4), [4,4.5), [4.5,5]; the permittivity range of the first layer ($\varepsilon_{1}\in \left[4,7\right]$) is divided into [4,4.6), [4.6,5.2), [5.2,5.8), [5.8,6.4), [6.4,7]; and the permittivity range of the second layer ($\varepsilon_{2}\in \left[5,8\right]$) is divided into [5,5.6), [5.6,6.2), [6.2,6.8), [6.8,7.4), [7.4,8]. In particular, for the three-layer pavement model, the thickness of the second layer is set to a random value between 8 cm and 12 cm, and the relative permittivity of the third layer is fixed at 4. It can be seen that the permittivity ranges of the first layer and the second layer are partially overlapped. In order to avoid insignificant parameter combinations (i.e., the permittivity of the first layer and second layer are equal), the following conditions must be met when combining these parameter intervals:

(19) $$y_{\left(l1,l2,l3\;\right)}=Label(\varepsilon_{1,l1},h_{1,l2},\varepsilon_{2,l3}),\{\begin{array}{c} 1\leq l1,l3\leq 5\\ 1\leq l2\leq 6\\ l1\leq l3 \end{array}$$

(20) $$y_{(l1,l2,l3)\;}=-3l_{1}^{2}+33l_{1}+l_{2}+6l_{3}-36$$

where, *l*1, *l*2, *l*3 are respectively the index numbers of the intervals in original ranges, and $y_{\left(l1,l2,l3\right)}$ is the number of the class. There are a total of 90 classes. For each class, 150 GPR data are respectively simulated by using the two-layer pavement models and the three-layer pavement models. Therefore, there are a total of 27,000 training data. To validate the classification performance of the MLPs, fifteen percent of the training data is used as the test set, and the test set would not be used to train the MLPs. In the numerical experiment, MLPs with two hidden layers are trained. There are 200 and 150 neurons in the two hidden layers, respectively. The number of neurons in the input layer and the output layer is respectively 216 and 90. The learning rate is in the form of exponential decay, with a base of 0.9 and an attenuation factor of 0.99. The training process of the MLPs is finished in the framework of TensorFlow. In the experiment, the MLPs are trained for 400 iterations. After each iteration, the trained MLPs are applied to the test data, and the classification accuracy is plotted versus the number of iterations in Figure 5. It can be found that the maximum classification accuracy is 98.6%, which means that the well trained MLPs can accurately classify the test data into corresponding classes according to the preset rule with a significantly high probability.

**Figure 6:**
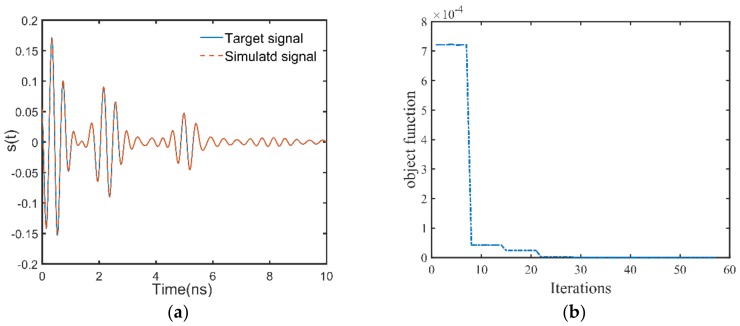
(**a**) Time domain waveforms of the GPR data simulated by the inversion results and the target signal. Good agreement can be seen between them. (**b**) The trajectory of the objective function during the inversion process. It can be seen that the objective function is only calculated by 30 times to find the global optimal solution.

To verify the performance of the hybrid method at the respect of full-waveform inversion, three GPR data are selected from the test set to perform full-waveform inversion, and two of which are correctly classified and one is misclassified. The two correctly classified GPR signals are respectively simulated by the two-layer pavement model and the three-layer pavement model. Since only the thickness of the first layer and the permittivities of the first two layers are estimated, the signals before 6.5 ns are matched when performing the full-waveform inversion. The correctly classified GPR data generated by the two-layer pavement model has the simulation parameter combination of $\varepsilon_{1}=4.493$, $h_{1}=2.41$, $\varepsilon_{2}=5.532$, and the corresponding class number of 1. According to the preset rule, the initial solution of the full-waveform inversion is $\varepsilon_{1}^{\mathrm{int}}=4.300$, $h_{1}^{\mathrm{int}}=2.25$, $\varepsilon_{2}^{\mathrm{int}}=5.300$. In addition, the antenna height is 0.462 m, which is considered as known in the full-waveform inversion. The antenna height is considered as known because it could be determined independently using other methods such as laser measurements in practical applications [8]. For the full-waveform inversion, the Levenberg-Marquardt (LM) algorithm is set with a maximum number of iterations of 70 and a step tolerance of $1\times 10^{-5}$. The inversion results are $\varepsilon_{1}^{\mathrm{inv}}=4.494$, $h_{1}^{inv}=2.41$, $\varepsilon_{2}^{inv}=5.535$, which agree well with the simulation parameter combination. Figure 6a shows the GPR data simulated by the inversion results and the target signal, and significant agreement can be seen between them. Figure 6b shows the trajectory of the objective function during the inversion process. It can be seen that the algorithm converges after only thirty iterations. According to the previous study on the topology structure of the inversion objective function [8], the function gradient is small in some ranges of the inversion parameters and large in other ranges. Therefore, in the inversion process, the objective function will show a tendency that it sometimes changes slowly and sometimes changes dramatically (Figure 6b).

**Figure 7:**
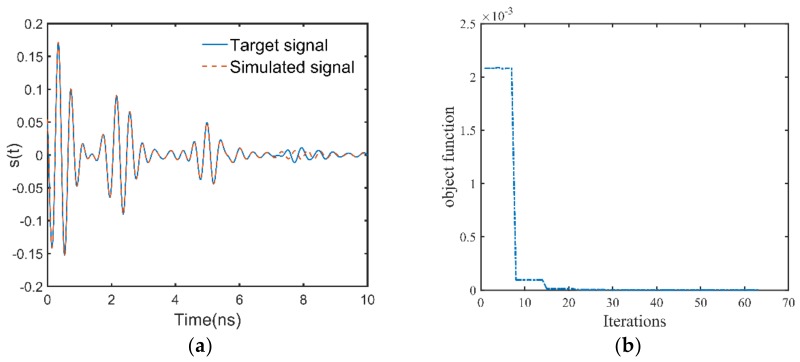
(**a**) Time domain waveforms of the GPR data simulated by the inversion results and the target signal. Since the time range for the inversion is within 6.5 ns, good agreement between the two waveforms can be seen before 6.5 ns. (**b**) The trajectory of the objective function during the inversion process.

**Figure 8:**
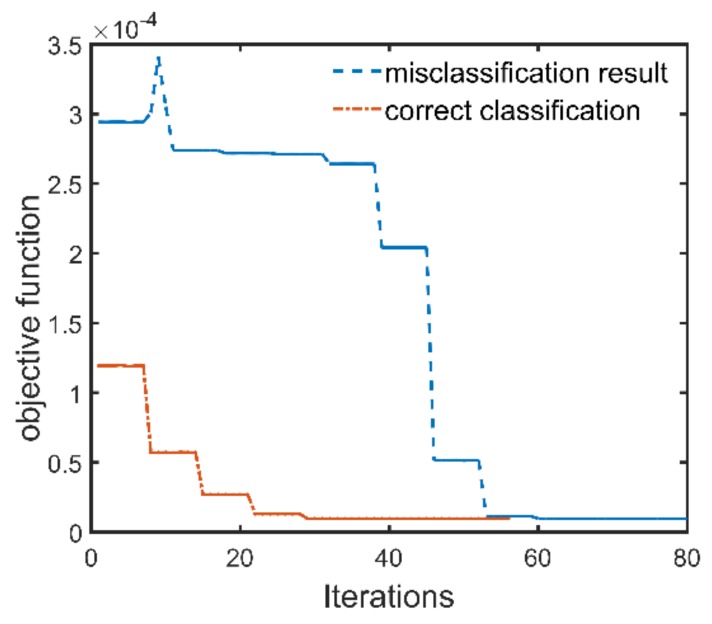
The trajectory of the objective function for the inversion of the misclassified GPR data. The initial solutions derived from the misclassification result and the correct classification result are used to perform the inversion, respectively. For the misclassification result, the LM algorithm converges after 60 iterations, and for the correct classification result, the LM algorithm converges after 30 iterations. In both cases, the LM algorithm converges fast.

**Figure 9:**
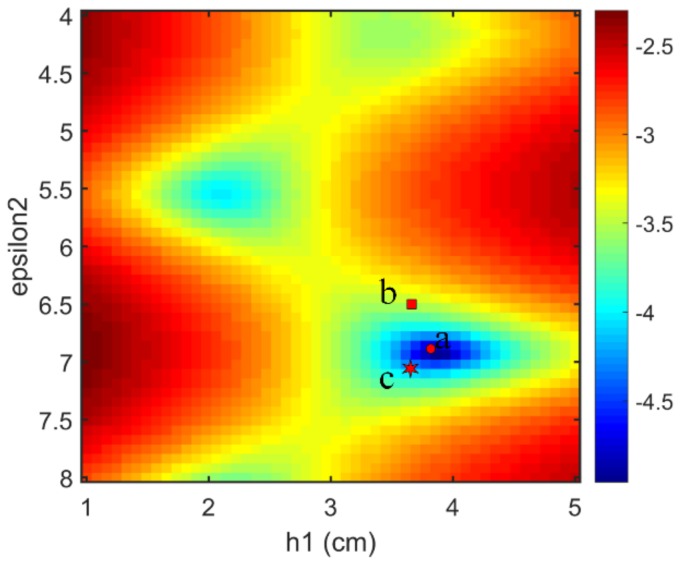
The 2-D response surface topographies of the objective functions in a logarithmic scale. (a) The circle denotes the global optimal solution. (b) The square denotes the initial solution derived from the misclassification result. (c) The star denotes the initial solution derived from the correct classification result.

The correctly classified GPR data generated by the three-layer pavement model has the simulation parameter combination of $\varepsilon_{1}=5.115$, $h_{1}=4.42$, $\varepsilon_{2}=7.992$, $h_{2}=11$, $\varepsilon_{3}=4$, and the corresponding class number of 53. Since only the thickness of the first layer and the permittivities of the first two layers are estimated, the time range for the inversion is within 6.5 ns, and the initial solution of the full-waveform inversion is $\varepsilon_{1}^{\mathrm{int}}=4.900$, $h_{1}^{\mathrm{int}}=4.25$, $\varepsilon_{2}^{\mathrm{int}}=7.700$. The antenna height is 0.460 m, and the configuration of the LM algorithm remains the same as before. The inversion results are $\varepsilon_{1}^{\mathrm{inv}}=5.112$, $h_{1}^{inv}=4.42$, $\varepsilon_{2}^{inv}=8.011$, which agree well with the simulation parameters of the target signal. Figure 7a shows the GPR signal simulated by the inversion results and the target signal, and significant agreement can be seen between them within 6.5 ns. Figure 7b shows the trajectory of the objective function during the inversion process. It can be found that the algorithm also converges after about thirty iterations. For the misclassified GPR data, it has the simulation parameter combination of $\varepsilon_{1}=5.680$, $h_{1}=3.92$, $\varepsilon_{2}=6.850$, $h_{2}=10$, $\varepsilon_{3}=4$, and the corresponding class number of 64. However, it is misclassified into class 58. According to the preset rule, the initial solution derived from the misclassification result is $\varepsilon_{1}^{\mathrm{int}}=5.500$, $h_{1}^{\mathrm{int}}=3.75$, $\varepsilon_{2}^{\mathrm{int}}=6.500$. Then, the initial solution is used for the full-waveform inversion, and the trajectory of the objective function during the inversion process is plotted in Figure 8. If this GPR data is correctly classified, its initial solution would be $\varepsilon_{1}^{\mathrm{int}}=5.500$, $h_{1}^{\mathrm{int}}=3.75$, $\varepsilon_{2}^{\mathrm{int}}=7.100$ according to the preset rule. This initial solution deduced by the correct classification result is also used for full-waveform inversion, and the trajectory of the objective function during the inversion process is also plotted in Figure 8. It can be seen from Figure 8 that the LM algorithm converges faster when the initial solution derived by the correct classification result is used. Nevertheless, the LM algorithm converges fast for both of the two initial solutions. The inversion results for both the two initial solutions are $\varepsilon_{1}^{\mathrm{inv}}=5.682$, $h_{1}^{inv}=3.92$, $\varepsilon_{2}^{inv}=6.859$, which agree well with the simulation parameters of the target signal. In order to understand the phenomenon in Figure 8, the 2-D response surface topography of the objective function is computed. The objective function is computed with $h_{1}$ and $\varepsilon_{2}$ as input. The value of $\varepsilon_{1}$ and the antenna height are considered as known. The misclassified test GPR data is the target signal of inversion. To better highlight the topography, the objective function values are express in a logarithmic scale (Figure 9). Figure 9 shows that several local minima are distributed around the global minimum, which is why the full-waveform inversion of the GPR data in the conventional methods require global optimization algorithms. The global minimum is in the center of a basin with a radius of 0.5 along the $\varepsilon_{2}$ direction and a radius of 1 cm along the $h_{1}$ direction. These radii are greater than half the lengths of the corresponding parameter intervals. This fact indicates that if a GPR data is correctly classified by the MLPs, the corresponding initial solution falls into the basin centered on the global optimal solution. Therefore, the gradient-based local optimization algorithm LM can quickly search from the initial solution to the global optimal solution. Since the MLPs extract a set of features from the training GPR data for each class, a newly input GPR signal is predicted to a class based on the degree of the similarity to these features. Because the features of the GPR data are determined by the characteristic parameters of the pavement, when a parameter of the GPR data is close to the parameter interval boundary, it may be misclassified to the nearest neighbor interval with a small probability. In this case, the distance between the initial solution and the global optimal solution would be slightly larger than the length of the half interval. Nevertheless, since the length of the parameter interval is relatively small, the initial solution of the misclassified GPR data will still falls within the basin centered on the global optimal solution. For example, the global optimal solution of the misclassified test GPR data is $opt=\left\{h_{1}=3.92,\varepsilon_{2}=6.850\right\}$, and the initial solution derived from the misclassification result is $\mathrm{mis}\_\mathrm{cls}=\left\{h_{1}^{\mathrm{int}}=3.75,\varepsilon_{2}^{\mathrm{int}}=6.500\right\}$. It can be seen from Figure 9 that the initial solution derived from the misclassification result still falls in the basin centered on the global optimal solution. If the above test data is correctly classified according to the experimental settings, the initial solution would be $cor\_cls=\left\{h_{1}^{\mathrm{int}}=3.75,\varepsilon_{2}^{\mathrm{int}}=7.100\right\}$. The distance between the initial solution derived from the misclassification result and the global optimal solution in the $\varepsilon_{2}$ direction is 0.35, and the distance between the initial solution derived from the correct classification result and the global optimal solution in the $\varepsilon_{2}$ direction is 0.25. Consequently, the misclassification result increases the distance between the initial solution and the optimal solution by 40%, and also increases the number of iterations of the inversion process.

The full-waveform inversion of these three test data have also been performed by conventional DE algorithm, which is the state-of-the-art method addressing the “thin layer problem”. The parameter search is performed on the corresponding entire range ($\varepsilon_{1}\in \left[4,7\right]$, $h_{1}\in [2,5]$, $\varepsilon_{2}\in \left[5,8\right]$), the number of the population is 10, and the maximum generation is 80 [10]. Table 1 shows the number of calculations of the objective function to find the global optimal solution. It can be seen from Table 1 that since the initial solution provided by the MLPs is close to the optimal solution, the optimal solution can be found after a small number of searches. The DE algorithm encounters many local minima in the inversion process, so a lot of trials are needed to find the global optimal value. Therefore, the hybrid method can improve the efficiency of the full waveform inversion.

### 3.2. Field Experiment

**Figure 10:**
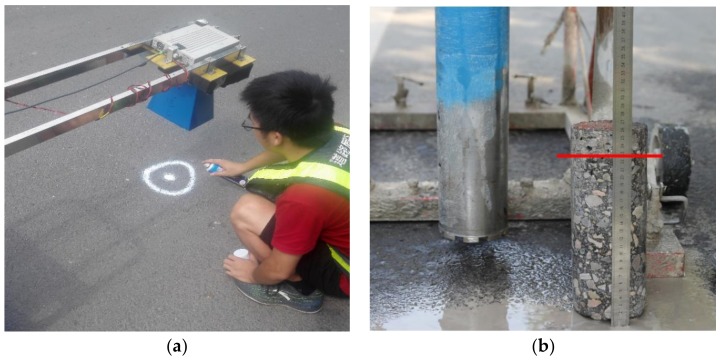
(**a**) Field experiment setup with the antenna height of 0.462 m. (**b**) Red line is the interface between the first layer and second layer, the measured thickness is 29 mm. The second layer and the third layer have a similar aggregate ratio, so that the reflection from the interface between them is weak.

**Figure 11:**
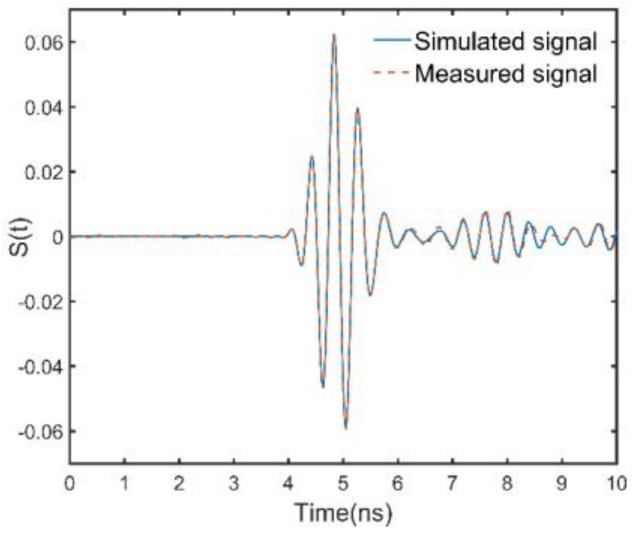
The measured signal and the signal simulated by the inversion results. Significant agreement between the measured signal and the simulated signal can be seen before 6.5 ns. Signals from 7 ns to 8 ns primarily consist of the multiple reflections from the pavement surface. The difference between the measured signal and the simulated signal at 9 ns is due to the reflection between the asphalt pavement and the lime-ash soil.

**Figure 12:**
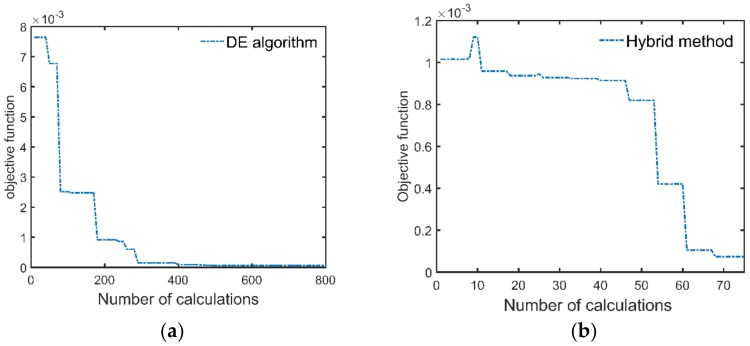
The number of calculations of the objective function during the full-waveform inversion. (**a**) Calculation number of the DE algorithm. (**b**) Calculation number of the Hybrid method. In order to find the global optimal solution, the objective function is respectively calculated by 500 times and 70 times for the DE algorithm and the hybrid method. Therefore, the hybrid method greatly improve the efficiency of the full-waveform inversion.

A field experiment is conducted with the GPR system at TIAN BEI road in Beijing (China). TIAN BEI road is an asphalt pavement which consists of three layers. The first layer of the pavement is the wear layer, its design thickness is 4 cm, and its aggregate size is less than 10 mm (AC-10). Due to the small size of the aggregate, the first layer can be regarded as a uniform layer, which is much in line with the pavement model assumption of the Lambot’s method. The design thicknesses of the second and the third layers are 10 cm and 12 cm, respectively. The second layer and the third layer have a similar aggregate ratio, which are composed with aggregate AC-16 and aggregate AC-23. Therefore, the echo from the interface between the second layer and the third layer is weak. Figure 10a shows the experimental setup. We use the GPR to perform A-scan above the road, and the antenna height is 0.46 cm. After the A scan, a field core of the pavement was drilled. Figure 10b is a core of the pavement. The red line in Figure 10b denotes the interface between the first layer and the second layer, and the measured thickness of the first layer was 29 mm. The waveform inversion of the corresponding GPR was performed by the means of DE algorithm and the hybrid method, respectively. Since the thickness of the first layer and the permittivities of the first two layers are estimated, only the signal before 6.5 ns is matched. The inversion results for both the methods are $\varepsilon_{1}=6.06$, $\varepsilon_{2}=6.89$, $h_{1}=30.2\;\mathrm{mm}$, and the thickness estimation error is 1.2 mm. Figure 11 shows the measured signal and the signal simulated by the inversion results. Significant agreement between the measured signal and the simulated signal can be seen before 6.5 ns. Signals from 7 ns to 8 ns primarily consist of the multiple reflections from the pavement surface and the echo from the interface between the second and the third layer. Since the echo from the interface between the second layer and the third layer is weak, the simulated signal and the measured signal agree well between 7 ns to 8 ns. The difference between the measured signal and the simulated signal at 9 ns is due to the reflection between the asphalt pavement and the lime-ash soil. Figure 12a shows the number of calculations of the objective function during the inversion process when using the DE algorithm. For the DE algorithm, the number of population is 10, so objective function would be calculated by 10 times in each generation. Figure 12b shows the number of calculations of the objective function of the hybrid method. It can be seen that the objective function is respectively calculated by 500 times and 70 times for the DE algorithm and the hybrid method to find the global optimal solution. Therefore, the hybrid method greatly improve the efficiency of the full-waveform inversion.

### 3.3. The Influence of Layer Thickness and Permittivity on results

As shown by the results in the Section 3.1, the performance of the proposed hybrid method depends on whether the MLPs can accurately classify the input GPR data. When the classification result of the MLPs is accurate, the initial solution derived from the classification result will be in the basin centered on the global optimal solution. In this case, the global optimal solution can be quickly searched by using the gradient-based local optimization algorithm. If GPR signal has a corresponding layer thickness and permittivity that exceeds the ranges of parameters used to train the MLPs, the MLPs cannot accurately classify the GPR signal. Therefore, the performance of the hybrid method will be affected. In the numerical experiment, the ranges of the layer thickness and permittivities used to train the MLPs are respectively $\varepsilon_{1}\in \left[4,7\right]$, $h_{1}\in [2\;\mathrm{cm},5\;\mathrm{cm}]$, $\varepsilon_{2}\in \left[5,8\right]$. We use two parameter combinations $S1=\left\{\varepsilon_{1}=3.40,h_{1}=2.30,\varepsilon_{2}=5.30\right\}$, $S2=\left\{\varepsilon_{1}=4.40,h_{1}=1.40,\varepsilon_{2}=5.70\right\}$ to simulate GPR data. For S1, the value of $\varepsilon_{1}$ exceeds 15% of the specified range, For S2, the value of $h_{1}$ exceeds 30% of the specified range. The GPR signals simulated by S1 and S2 are sent to the hybrid method. Table 2 shows the inversion results of these two GPR signals.

It can be seen from Table 2 that since the parameters of S1 and S2 are beyond the specified parameter ranges to a large extent (more than 15%), the initial solution derived from the classification result of MLPs is far from the global optimal solution, so the estimation errors of the hybrid method are great. In order to accurately invert the parameters of S1 and S2, we adjust the training parameter ranges of the MLPs to $\varepsilon_{1}\in \left[3,6\right]$, $h_{1}\in [1\;\mathrm{cm},4\;\mathrm{cm}]$, $\varepsilon_{2}\in \left[4,7\right]$. These parameter ranges are divided into small intervals according to the same operations in Section 3.1. The interval lengths of $\varepsilon_{1}$ and $\varepsilon_{2}$ are 0.6, and the interval length of $h_{1}$ is 0.5 cm. The MLPs uses the 27,000 GPR data generated over these parameter intervals for training, with a highest classification of 98.7%. The GPR signals simulated by S1 and S2 are again sent to the hybrid method. Table 3 shows the inversion results of S1 and S2 after the training parameter ranges adjustment. It can be seen from the results in Table 3 that when the ranges of the training parameters of the MLPs is adjusted to cover the parameters of S1 and S2, the parameters of S1 and S2 can be accurately inverted using the hybrid method. Combining the results of Table 2 and Table 3, it can be seen that the minimum layer thickness and permittivity that can be successfully detected by the hybrid method depend on the ranges of training parameters of the MLPs. In pavement inspection, the training parameter ranges of MLPs can be set to cover the possible properties values, so the hybrid method will have good performance.

Just like the hybrid method, the state-of-the-art full-waveform inversion method based on DE algorithm is also affected by the ranges of search parameters set. We compared the performance of the two methods in case where the characteristics corresponding to the GPR data are slightly outside the training parameter ranges of the hybrid method and the parameter search ranges of the DE inversion method, for example, within 5%. We used other two parameter combinations $S3=\left\{\varepsilon_{1}=3.80,h_{1}=2.30,\varepsilon_{2}=5.20\right\}$ and $S4=\left\{\varepsilon_{1}=4.40,h_{1}=1.90,\varepsilon_{2}=5.70\right\}$ to simulate GPR data, and the training parameter ranges of the hybrid method and parameter search ranges of the DE method are same ($\varepsilon_{1}\in \left[4,7\right]$, $h_{1}\in [2\;\mathrm{cm},5\;\mathrm{cm}]$, $\varepsilon_{2}\in \left[5,8\right]$). For S3 and S4, the values of $\varepsilon_{1}$ and $h_{1}$ exceed 5% of the specified ranges. Table 4 shows the inversion results of the hybrid method and the DE inversion method for S3 and S4 in case where the parameters values are slightly outside the specified ranges. The results show that the hybrid method can accurately invert these parameters, and the DE inversion method has a maximum thickness estimation error of 0.12 cm and permittivity estimation error of 0.43.

## 4. Conclusions

In this article, a hybrid method based on the MLPs and the Levenberg-Marquardt algorithm is applied to improve the efficiency of the full-waveform inversion of the GPR data. The MLPs network provides an initial solution of the full-waveform inversion for the input GPR data according to the preset rule. Then, the Levenberg-Marquardt algorithm is used to perform a more accurate inversion of the input GPR signal from the initial solution. The results of the numerical experiment and field experiment show that the hybrid method greatly improves the efficiency of the full-waveform inversion, and the inversion results of the hybrid method has the same accuracy with that of the state-of-the-art method. The minimum layer thickness and permittivity that can be successfully detected by the hybrid method depend on the ranges of training parameters of the MLPs. When the parameters values to be detected are slightly outside the specified ranges, the hybrid method has better performance than the state-of-the-art full-waveform inversion method. Although the feasibility of this hybrid method is verified in a thin layer case, it can be easily extended to other situations by adding corresponding training data. Feature work would study more efficient methods to provide initial solution for the full-waveform inversion.

## Figures and Tables

**Table 1:** The number of calculations of the objective function before convergence for the three test data.

	Correct Classification		Misclassification
Algorithms	Two Layer Data	Three Layer Data	Three Layer Data
Hybrid method	30	30	60
DE	410	440	450

**Table 2:** The inversion results of the hybrid method for GPR signals simulated by parameter combinations S1 and S2. For S1, the value of $\varepsilon_{1}$ exceeds 15% of the specified range, for S2, the value of $h_{1}$ exceeds 30% of the specified range.

Parameters to Be Detected	Classification Result	Initial Solution	Inversion Results	Estimation Errors
$\;[\varepsilon_{1}=3.40$ $\;h_{1}=2.30$ $\;\varepsilon_{2}=5.30]$	16	$\;[\varepsilon_{1}^{int}=4.30$ $\;h_{1}^{int}=3.75$ $\;\varepsilon_{2}^{int}=6.50]$	$\;[\varepsilon_{1}^{inv}=2.65$ $\;h_{1}^{inv}=13.21$ $\;\varepsilon_{2}^{inv}=2.55]$	$\;[\Delta \varepsilon_{1}=0.75$ $\;\Delta h_{1}=10.01$ $\;\Delta \varepsilon_{2}=2.75]$
$\;[\varepsilon_{1}=4.40$ $\;h_{1}=1.40$ $\;\varepsilon_{2}=5.70]$	34	$\;[\varepsilon_{1}^{int}=4.90$ $\;h_{1}^{int}=3.75$ $\;\varepsilon_{2}^{int}=5.90]$	$\;[\varepsilon_{1}^{inv}=3.63$ $\;h_{1}^{inv}=4.88$ $\;\varepsilon_{2}^{inv}=4.09]$	$\;[\Delta \varepsilon_{1}=0.77$ $\;\Delta h_{1}=3.48$ $\;\Delta \varepsilon_{2}=1.61]$

**Table 3:** The inversion results of S1 and S2 after the training parameter ranges adjustment. The training parameter ranges of the MLPs are adjusted into $\varepsilon_{1}\in \left[3,6\right]$, $h_{1}\in [1\;\mathrm{cm},4\;\mathrm{cm}]$, $\varepsilon_{2}\in \left[4,7\right]$.

Parameters to Be Detected	Classification Result	Initial Solution	Inversion Results	Estimation Errors
$\;[\varepsilon_{1}=3.40$ $\;h_{1}=2.30$ $\;\varepsilon_{2}=5.30]$	15	$\;[\varepsilon_{1}^{int}=3.30$ $\;h_{1}^{int}=2.25$ $\;\varepsilon_{2}^{int}=5.50]$	$\;[\varepsilon_{1}^{inv}=3.40$ $\;h_{1}^{inv}=2.30$ $\;\varepsilon_{2}^{inv}=5.30]$	$\;[\Delta \varepsilon_{1}=0.00$ $\;\Delta h_{1}=0.00$ $\;\Delta \varepsilon_{2}=0.00]$
$\;[\varepsilon_{1}=4.40$ $\;h_{1}=1.40$ $\;\varepsilon_{2}=5.70]$	55	$\;[\varepsilon_{1}^{int}=4.50$ $\;h_{1}^{int}=1.25$ $\;\varepsilon_{2}^{int}=5.50]$	$\;[\varepsilon_{1}^{inv}=4.40$ $\;h_{1}^{inv}=1.40$ $\;\varepsilon_{2}^{inv}=5.71]$	$\;[\Delta \varepsilon_{1}=0.00$ $\;\Delta h_{1}=0.00$ $\;\Delta \varepsilon_{2}=0.01]$

**Table 4:** The inversion results of the hybrid method and the DE inversion method for S3 and S4 in case where the parameters values are slightly outside the specified ranges. For S3 and S4, the values of $\varepsilon_{1}$ and $h_{1}$ exceed 5% of the specified ranges.

Parameters to Be Detected	Inversion Results for Hybrid Method	Estimation Errors for Hybrid Method	Inversion Results for DE Method	Estimation Errors for DE Method
$\;[\varepsilon_{1}=3.80$ $\;h_{1}=2.30$ $\;\varepsilon_{2}=5.20]$	$\;[\varepsilon_{1}^{inv}=3.80$ $\;h_{1}^{inv}=2.30$ $\;\varepsilon_{2}^{inv}=5.20]$	$\;[\Delta \varepsilon_{1}=0.00$ $\;\Delta h_{1}=0.00$ $\;\Delta \varepsilon_{2}=0.00]$	$\;[\varepsilon_{1}^{inv}=4.00$ $\;h_{1}^{inv}=2.18$ $\;\varepsilon_{2}^{inv}=5.63]$	$\;[\Delta \varepsilon_{1}=0.20$ $\;\Delta h_{1}=0.12$ $\;\Delta \varepsilon_{2}=0.43]$
$\;[\varepsilon_{1}=4.40$ $\;h_{1}=1.90$ $\;\varepsilon_{2}=5.70]$	$\;[\varepsilon_{1}^{inv}=4.40$ $\;h_{1}^{inv}=1.90$ $\;\varepsilon_{2}^{inv}=5.70]$	$\;[\Delta \varepsilon_{1}=0.00$ $\;\Delta h_{1}=0.00$ $\;\Delta \varepsilon_{2}=0.00]$	$\;[\varepsilon_{1}^{inv}=4.23$ $\;h_{1}^{inv}=2.00$ $\;\varepsilon_{2}^{inv}=5.33]$	$\;[\Delta \varepsilon_{1}=0.17$ $\;\Delta h_{1}=0.10$ $\;\Delta \varepsilon_{2}=0.37]$
